# Supplementary material for: Establishing an Ex Vivo Culture Model of Human Proximal Airway Tissue
Source: Methods Protoc. 2025 Nov 2;8(6):132. doi: 10.3390/mps8060132 (PMC12641672; doi:10.3390/mps8060132)
Supplement: Supplementary file 1 [file mps-08-00132-s001.zip › mps-3874311-supplementary.pdf]

SUPPLEMENTARY MATERIAL:

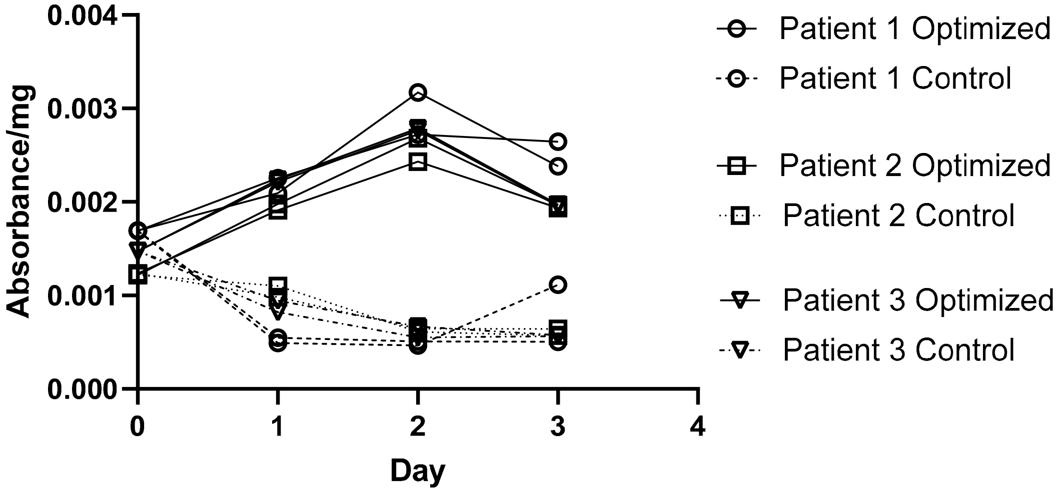

Figure S1. Individual data points for TTC labeling for each patient as extension of Figure 1C.

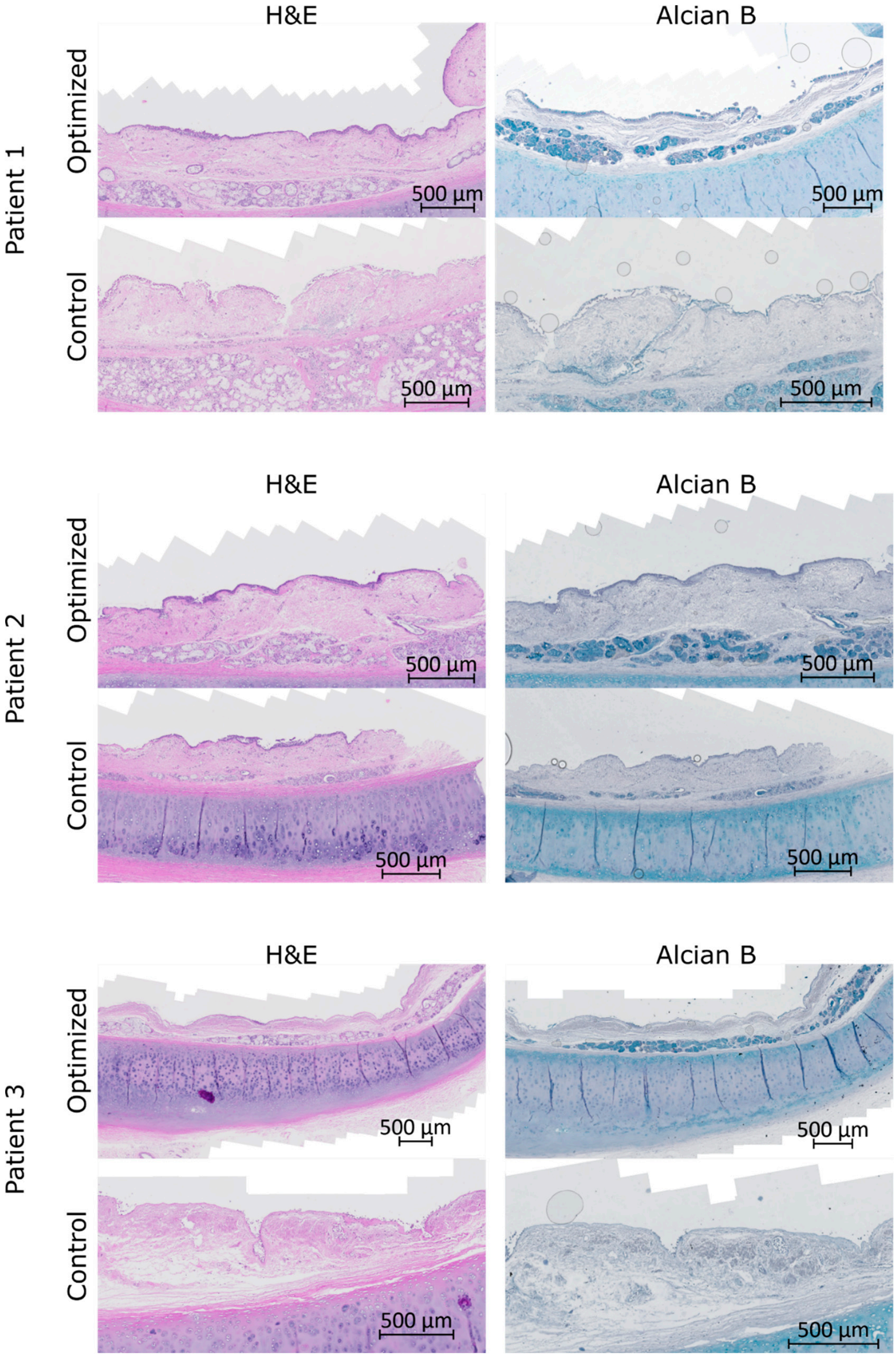
H&E
Alcian B
Patient 1
Optimized
Control
500 µm
Patient 2
Patient 3

Figure S2. Extension of Fig 2 A-B showing larger (~2mm) section H&E and AlcianB stained images of the cultured tissue samples for control and optimized models of 3 patient samples. The optimized tissues show better retainment of the epithelial layers compared to control cultures.

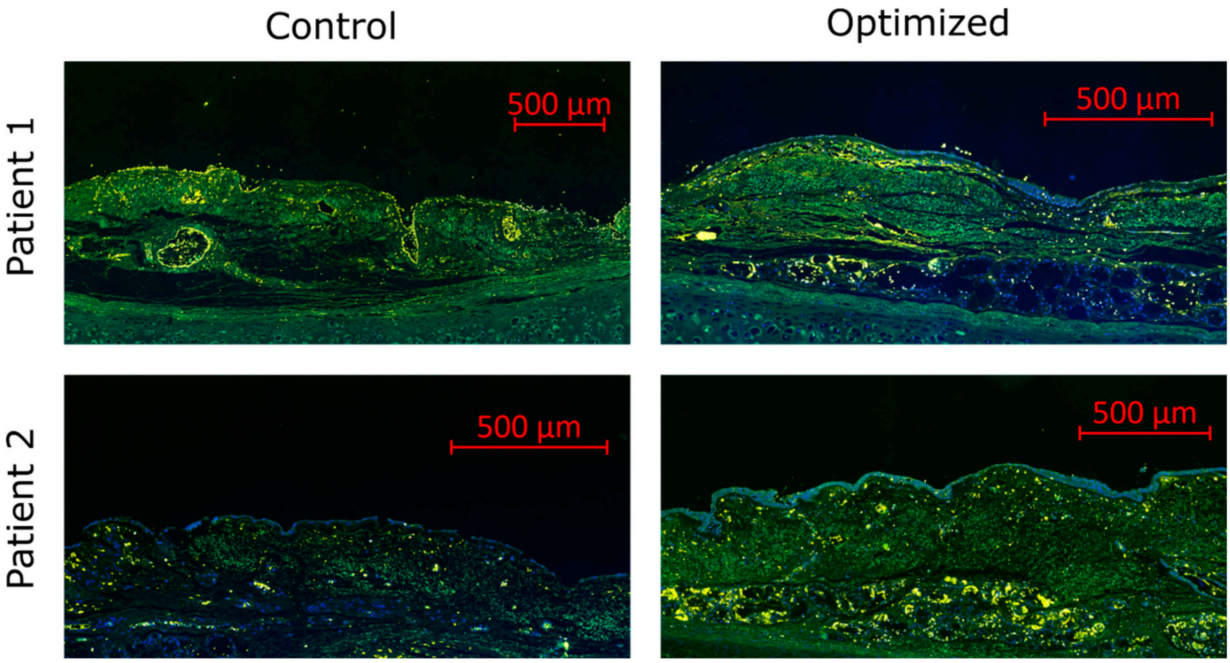

Figure S3. Extension of Fig 2C showing low magnification fields for sections of TUNEL images of the cultured tissue samples for control and optimized models of two patient samples. The optimized tissues show better retainment of the epithelial layers (blue) compared to control.

Azide→biotin Western blot

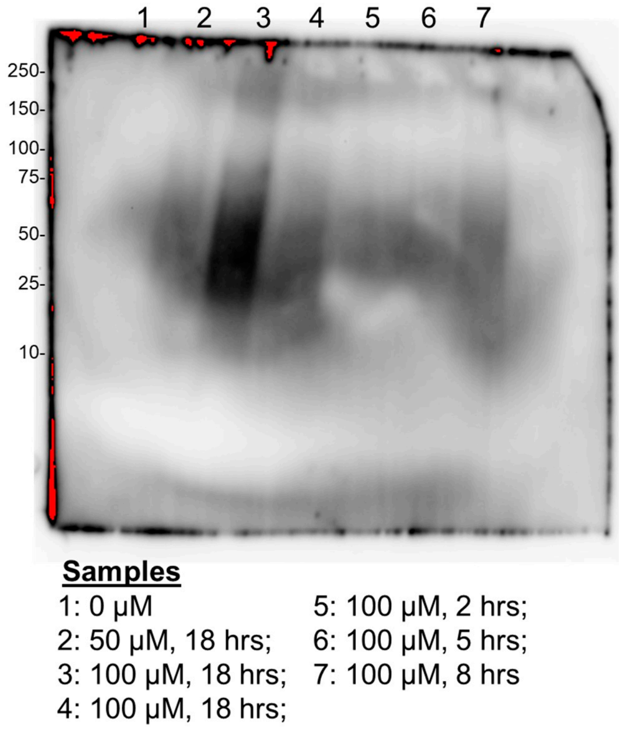

Samples

1: 0 μM
2: 50 μM, 18 hrs;
3: 100 μM, 18 hrs;
4: 100 μM, 18 hrs;
5: 100 μM, 2 hrs;
6: 100 μM, 5 hrs;
7: 100 μM, 8 hrs

Figure S4. The supplemental figure for Fig 3B shows a whole gel image of western blotting. The western blotting was performed to detect the azide labeled proteins by metabolic labeling using AcGalNAz in ex vivo airway culture system and showed the probe dose-dependent response and time dependent changes in the labeled proteins in human proximal airways.
